# SARS-CoV-2 Disease Severity in the Golden Syrian Hamster Model of Infection Is Related to the Volume of Intranasal Inoculum

**DOI:** 10.3390/v15030748

**Published:** 2023-03-14

**Authors:** Alastair Handley, Kathryn A. Ryan, Elizabeth R. Davies, Kevin R. Bewley, Oliver T. Carnell, Amy Challis, Naomi S. Coombes, Susan A. Fotheringham, Karen E. Gooch, Michael Charlton, Debbie J. Harris, Chelsea Kennard, Didier Ngabo, Thomas M. Weldon, Francisco J. Salguero, Simon G. P. Funnell, Yper Hall

**Affiliations:** 1 UKHSA Porton, Vaccine Development and Evaluation Centre, UK Health Security Agency, Manor Farm Road, Salisbury SP4 0JG, UK; 2 Quadram Institute Bioscience, Norwich Research Park, Norwich NR4 7UQ, UK; 3 World Health Organization, Appia 20, 1211 Geneva, Switzerland

**Keywords:** SARS-CoV-2, Syrian hamster, animal model, coronavirus

## Abstract

The golden Syrian hamster (*Mesocricetus auratus*) is now commonly used in preclinical research for the study of severe acute respiratory syndrome coronavirus 2 (SARS-CoV-2) infection and the assessment of vaccines, drugs and therapeutics. Here, we show that hamsters inoculated via the intranasal route with the same infectious virus dose of prototypical SARS-CoV-2 administered in a different volume present with different clinical signs, weight loss and viral shedding, with a reduced volume resulting in reduced severity of disease similar to that obtained by a 500-fold reduction in the challenge dose. The tissue burden of the virus and the severity of pulmonary pathology were also significantly affected by different challenge inoculum volumes. These findings suggest that a direct comparison between the severity of SARS-CoV-2 variants or studies assessing the efficacy of treatments determined by hamster studies cannot be made unless both the challenge dose and inoculation volume are matched when using the intranasal route. Additionally, analysis of sub-genomic and total genomic RNA PCR data demonstrated no link between sub-genomic and live viral titres and that sub-genomic analyses do not provide any information beyond that provided by more sensitive total genomic PCR.

## 1. Introduction

SARS-CoV-2 was first identified in the lower respiratory tract of patients presenting with viral pneumonia in December 2019, before triggering a pandemic declaration by the World Health Organisation on the 11 March 2020 [1]. The disease (COVID-19) which results from infection with SARS-CoV-2 virus in humans causes a broad spectrum of respiratory symptoms, from very mild to severe, life-threatening and fatal illness. Fatality and severe disease have been shown to be associated with human host risk factors including obesity and elderly ages [2]. An unprecedented global effort was initiated early in the pandemic to develop drugs, vaccines and therapeutics to prevent death, aid treatment and prevent the spread of COVID-19. With new SARS-CoV-2 variants continuing to arise, continuous research and development are needed to ensure vaccines and therapeutics remain effective.

The golden Syrian hamster has become the species of choice for in vivo preclinical assessment of virulence of variants of SARS-CoV-2 and preclinical research assessing the efficacy of vaccines, antivirals and therapeutics. The hamster experiences mild to severe disease with measurable clinical signs, significant weight loss, viral shedding, lung pathology and immune response after intranasal (IN) inoculation with SARS-CoV-2 virus [3]. Unlike other animal species such as ferrets [4,5] and non-human primates [6], which usually develop mild disease, the wide range of quantitative and qualitative outputs provided by the hamster intranasal infection model allows for sufficient discriminatory power to measure the efficacy of vaccines, drugs or therapeutics against SARS-CoV-2 [7].

Published reports comparing BA.1 and BA.2 variants of SARS-CoV-2 in the hamster model of IN infection have produced different results in relation to the severity of the resulting disease. For example, Yamasoba et al. [8] found SARS-CoV-2 variant Omicron BA.2 caused more severe disease than the BA.1 variant after inoculation using a volume of 100 µL (1 × 10^4^ TCID50/mL dose), while Kawaoka et al. [9] found both variants similarly pathogenic after inoculation using a volume of 30 µL (both 1 × 10^3^ and 1 × 10^5^ plaque forming units (PFU)/mL dosages used).

There is currently no widely accepted standard method for the IN challenge inoculation dose and volume beyond the maximum volumes acceptable for animal welfare standards. This has resulted in a wide variance in volumes used between different laboratories. In many published results, there is a lack of specific information such as whether inoculation volumes refer to the total inoculation volume or volume given per nare, how the hamsters were restrained and whether or not anaesthesia or sedation was used (Table 1). This lack of specific information about volume and administration is highly likely to compromise the ability to compare findings between studies.

In this study, when we challenged hamsters with the same infectious virus dose administered in inoculation with a different (four-fold) inoculum volume, we observed different disease severity outcomes. Fifty microlitres (25 µL per nare) resulted in mild disease but two hundred microlitres (100 µL per nare) resulted in moderate disease. 

In our further studies, we found that in order to achieve a similar difference in severity using the same volume (200 µL) we needed to use a 500-fold dilution of live virus. These findings suggest that to compare different SARS-CoV-2 variants and assess the efficacy of vaccines, drugs and therapeutics, the same volume of the challenge as well as the viral dose in the inoculum should be used.

## 2. Materials and Methods

**Viruses and Cells:** SARS-CoV-2 Australia/VIC01/202035 was generously provided by The Peter Doherty Institute, Melbourne, Australia at P1 after primary growth in Vero/hSLAM cells and subsequently passaged twice at UKHSA Porton in Vero/hSLAM cells [ECACC 04091501]. Infection of cells was with ~0.0005 MOI of virus and harvested at day 4 by dissociation of the remaining attached cells by gentle rocking with sterile 5 mm borosilicate beads followed by clarification by centrifugation at 1000× *g* for 10 min. Whole genome sequencing was performed, on the P3 challenge stock, using SISPA amplification on both Nanopore and Illumina technologies as described previously [18]. The virus titre of the VIC01 challenge stocks was determined by plaque assay on Vero/E6 cells [ECACC 85020206]. Cell lines were obtained from the European Collection of Authenticated Cell Cultures (ECACC) UKHSA, Porton Down, UK. Cell cultures were maintained at 37 °C in MEM (Life Technologies, Carlsbad, CA, USA) supplemented with 10% foetal bovine serum (Sigma, Hertfordshire, UK) and 25 mM HEPES (Gibco, Paisley, UK), 2 mM L-Glutamine (Gibco), 1× non-essential amino acids solution (Gibco). In addition, Vero/hSLAM cultures were supplemented with 0.4 mg/mL of geneticin (Invitrogen, Oxford, UK) to maintain the expression plasmid.

**Animals:** Twelve healthy, golden Syrian hamsters (*Mesocricetus auratus*) were obtained from a UK Home Office accredited supplier (Envigo RMS, Oxon, UK). Animals were housed individually at the Advisory Committee on Dangerous Pathogens (ACDP) containment level 3. Cages met with the UK Home Office Code of Practice for the Housing and Care of Animals Bred, Supplied or Used for Scientific Procedures (December 2014). Access to food and water was ad libitum and environmental enrichment was provided. All experimental work was conducted under the authority of and in compliance with a UK Home Office approved project licence that had been subject to local ethical review at UKHSA Porton Down by the Animal Welfare and Ethical Review Body (AWERB) as required by the Home Office Animals (Scientific Procedures) Act 1986. A second set of six hamsters originally used in a separate study were included for comparative purposes [14].

**Experimental Design:** Before the start of the experiment, animals were randomly assigned to challenge groups to minimise bias. An identifier chip (Plexx IPTT-300 temperature transponder) was inserted subcutaneously into each animal. Prior to the challenge, animals were sedated by isoflurane. The challenge virus was delivered by intranasal instillation (200 µL total, 100 µL per nostril for the standard volume inoculation and 50 µL total, 25 µL per nostril for the reduced volume inoculation) diluted in phosphate-buffered saline (PBS). During intranasal inoculation, the hamsters are held in a ventral dorsal decubitus position with the head forming a 45° angle. The intranasal administration procedure is performed slowly, drop by drop, to ensure each aliquot of inoculum has entered the nasal cavity before the next drop is administered. The standard target dose of 5 × 10^4^ PFU/mL VIC01 was delivered to both groups (*n* = 6) of hamsters. Hamsters were throat swabbed at days 2, 4, 6 and 7 post-challenge. A second set of one group (*n* = 6) of hamsters was included from a previous study, receiving the standard volume inoculation and a reduced dose of 1 × 10^2^ PFU/mL. These hamsters were throat swabbed at days 2, 4, 6 and 8.

**Clinical observations:** Hamsters were monitored for temperature via Plexx IPTT-300 temperature transponders and for clinical signs of disease twice daily (approximately 8 h apart). Clinical signs of disease were assigned a score based upon the following criteria (score in brackets): healthy (0), lethargy (1), behavioural change (1), sunken eyes (2), ruffled (2), wasp-waisted (3), dehydrated (3), arched (3), coughing (3), laboured breathing 1-occasional catch or skip in breathing rate (5) and laboured breathing 2-abdominal effort with breathing difficulties (7). Animals were weighed at the same time each day until euthanasia. 

**Post-mortem examination and pathology.** Hamsters were given an anaesthetic overdose (sodium pentabarbitone Dolethal, Vetquinol UK Ltd., Titchmarsh, UK, 140 mg/kg) via intraperitoneal injection and exsanguination was effected via cardiac puncture. A necropsy was performed immediately after confirmation of death. Each animal was assigned a histology ID number for blinding purposes. Lung (left lobe and caudal right lobe) and nasal cavity samples were fixed in neutral buffered formalin. The nasal cavity was decalcified using an EDTA solution prior to embedding in paraffin wax. Tissue sections were stained with haematoxylin and eosin (H&E) and scanned by a Hamamatsu NanoZoomer S360 and viewed with NDP.view2 software (v2.9.29). The pathologist was blinded to treatment and group details and the slides were randomised prior to examination in order to prevent bias (blind evaluation). 

A semi-quantitative, subjective scoring system is used to evaluate the severity of lesions observed in the lung and nasal cavity [19]. Additionally, the percentage of area comprising pneumonia in the lung was calculated using digital image analysis (Nikon-NIS-Ar, Nikon UK, Surrey, UK). RNAscope (an in situ hybridisation method used on formalin-fixed, paraffin-embedded tissues) was used to identify the SARS-CoV-2 virus in all tissues. Briefly, tissues were pre-treated with hydrogen peroxide for 10 min (RT), target retrieval for 15 min (98–101 °C) and protease plus for 30 min (40 °C) (all Advanced Cell Diagnostics, Abingdon, UK). A V-nCoV2019-S probe (Advanced Cell Diagnostics) targeting the S-protein gene was incubated on the tissues for 2 h at 40 °C. Amplification of the signal was carried out following the RNAscope protocol (RNAscope 2.5 HD Detection Reagent–Red) using the RNAscope 2.5 HD red kit (Advanced Cell Diagnostics). RNAScope stained sections were also scanned and digital image analysis was carried out in order to calculate the total area of the lung section positive for viral RNA. For the nasal cavity, a semiquantitative scoring system was applied to evaluate the presence of virus RNA: 0 = no positive staining; 1 = minimal; 2 = mild; 3 = moderate and 4 = abundant staining.

**RNA Extraction:** Throat swabs were inactivated in AVL plus ethanol and RNA was isolated. Downstream extraction was performed using the BioSprint™96 One-For-All vet kit (Indical, Leipzig, Germany) and Kingfisher Flex platform as per the manufacturer’s instructions. 

**Quantification of Viral RNA by RT-qPCR:** Reverse transcription-quantitative polymerase chain reaction (RT-qPCR) targeting a region of the SARS-CoV-2 nucleocapsid (N) gene was used to determine viral loads and was performed as previously described [4]. Positive swab and fluid samples detected below the limit of quantification (LLOQ) of 12,857 copies mL were assigned the value of 5 copies/µL, which equates to 6429 copies/mL, whilst undetected samples were assigned the value of <2.3 copies/µL, equivalent to the assay’s lower limit of detection (LLOD), which equates to 2957 copies/mL. Additional PCR data was taken from a separate, previous study for analysis of the relationship between live viral titre, total genomic RNA titre and sub-genomic RNA titre. Samples were processed and analysed using the same methods listed here.

**Confirmation of Challenge Dose by Plaque Assay:** The challenge dose was confirmed by plaque assay prepared on the day of challenge. Dilutions of the challenge material were plated in triplicate on each assay plate for confirmation of the challenge titre. The challenge dose was diluted in MEM containing added antibiotic/antimycotic (Life Technologies) and no serum and incubated in 24-well plates (Nunc, ThermoFisher Scientific, Loughborough, UK) with Vero E6 cell monolayers. The virus was allowed to adsorb at 37 °C for 1 h, then overlaid with MEM containing 1.5% carboxymethylcellulose (Sigma), 4% (*v*/*v*) foetal bovine serum (Sigma) and 25 mM HEPES buffer (Life Technologies). After incubation at 37 °C for 5 days, the plates were fixed overnight with 20% (*w*/*v*) formalin/PBS, washed with tap water and stained with methanol crystal violet solution (0.2% *w*/*v*) (Sigma).

**Focus forming assay (FFA):** The virus titre for live SARS-CoV-2 throat swab samples was determined by focus forming assay on Vero/E6 cells [ECACC 85020206]. Ninety-six well plates were seeded with 2.5 × 10^4^ cells/well the day prior to infection and then washed twice with Dulbecco’s PBS (DPBS). Ten-fold serial dilutions (1 × 10^−1^ to 1 × 10^−6^) of virus stocks were prepared in MEM (supplemented with 25 mM HEPES (Gibco), 2 mM L-Glutamine (Gibco), 1× non-essential amino acids solution (Gibco)). A hundred microliter virus inoculum was added per well in duplicate and incubated for 1 h at 37 °C. The virus inoculum was removed, and cells overlaid with MEM containing 1% carboxymethylcellulose (Sigma), 4% (*v*/*v*) heat-inactivated foetal calf serum (FCS) (Sigma), 25 mM HEPES buffer (Gibco), 2 mM L-Glutamine (Gibco), 1× non-essential amino acids solution (Gibco). After incubation at 37 °C for 26 h, cells were fixed overnight with 8% (*w*/*v*) formalin/PBS, washed with water and permeabilised with 0.2% (*w*/*v*) Triton X-100/PBS at room temperature for 10 min. Cells were washed with PBS, incubated with 0.3% hydrogen peroxide (Sigma) at room temperature for 20 min and washed with PBS. Foci were stained with 50 µL/well rabbit anti-nucleocapsid (Sino Biological, 40588-T62) diluted 1:1000 in 0.2% (*w*/*v*) Triton X-100/PBS for 1 h at room temperature. Antibody dilutions were discarded and cells were washed with PBS and incubated with 50 µL/well goat anti-rabbit IgG HRP (Invitrogen, G-21234) diluted 1:4000 in 0.2% (*w*/*v*) Triton X-100/PBS for 1 h at room temperature. Cells were washed with PBS and incubated with TrueBlue peroxidase substrate (SeraCare, 5510-0030) for 10 min at room temperature then washed with water. Infectious foci were counted with an ImmunoSpot^®^ S6 Ultra-V analyser with BioSpot counting module (Cellular Technologies Europe). Titre (FFU/mL) was determined by the following formula: titre (FFU/mL) = No. of foci/(Dilution factor × 0.1).

**Statistical Analysis:** Statistical analysis was performed on Log10 transformed PCR data using R version 4.1.3. To compare group throat swab viral loads post-challenge with their respective re-challenge viral loads, data were analysed using Tukey’s honest significant difference (HSD)-corrected pairwise multiple comparisons mixed-effects analysis of variance (ANOVA). The fixed effects in the model were sample days post challenge (DPC) and the group and the random effect was the individual animal. Culls were performed at 7 DPC and 35 DPC (groups 1 and 5 at 7 DPC and groups 2 to 4 at 35 DPC). Viral RNA copies in collected lung tissue from each group were compared by ANOVA with post hoc pairwise Tukey’s HSD to compare viral RNA measured in different groups and DPC. Weight percentage change data were also analysed by Tukey-corrected pairwise multiple comparison ANOVA. Parametric statistical analyses were selected as the data were expected to conform to a log-normal distribution (for qPCR results) or a normal distribution (for weights) based on historical observations of data from similar hamster challenge studies. Ordinal clinical score data were analysed using a non-parametric (Kruskal–Wallis) test. Histopathological results were analysed by a Mann–Whitney’s U test. No statistical analysis was undertaken on temperature data as no trends were observed.

## 3. Results

**Study Design:** Hamsters (*n* = 6 per group with an equal male/female ratio) were challenged intranasally with Australia/VIC01/202035 SARS-CoV-2 (VIC01), in two volumes, a 200 μL or 50 μL volume at the standard dose (5 × 10^4^ PFU). A further group was challenged using a 200 μL inoculum volume at a reduced (500-fold less) viral dose (1 × 10^2^ PFU) and not culled during the study timeframe. Groups inoculated with the standard viral dose in 200 μL and in 50 μL were culled 7 days after challenge for evaluation of lung disease severity. Plaque forming unit (PFU) back-titration was completed on the day of the challenge on samples of the inoculum, confirming the target dose of SARS-CoV-2 was administered to each group.

**Figure 1:**
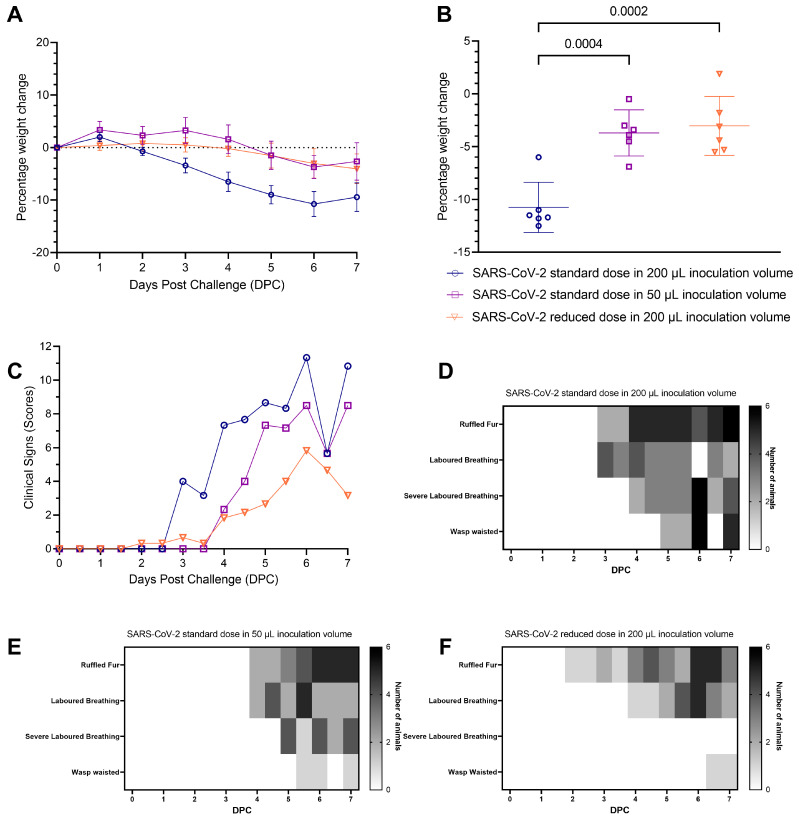
Clinical observations of pathogenicity in hamsters challenged with SARS-CoV-2 delivered in varied inoculum volumes and viral titres. Standard dose of SARS-CoV-2 is 5 × 10^4^ Plaque forming units (PFU)/mL, Reduced dose is 1 × 10^2^ PFU/mL. (**A**) Percentage weight change from weight at day of challenge measured daily. Lines indicate geomeans and bars indicate 95% confidence interval. (**B**) Weight change at 6 Days Post Challenge (DPC) as a percentage of the initial weight at the day of challenge. Lines indicate means and bars indicate 95% confidence interval. Statistical analysis by one-way ANOVA with Tukey’s multiple comparison correction (*p* < 0.05 displayed). (**C**) Clinical scores of the hamsters measured daily, each point is median of six animals, there was high variability and as such error bars are not displayed to improve clarity. Scores were assigned using the following scoring system: Ruffled Fur = 2, Wasp waisted = 3, Laboured breathing = 5, Severe Laboured Breathing = 7. (**D**) Number of hamsters displaying each clinical sign per day in hamsters inoculated with the standard dose in 200 μL. (**E**) Number of hamsters displaying each clinical sign per day in hamsters inoculated with the standard dose in 50 μL. (**F**) Number of hamsters displaying each clinical sign per day in hamsters inoculated with the reduced dose in 200 μL.

A lower challenge inoculation volume produced milder clinical signs of infection and respiratory pathology in hamsters compared to a higher inoculation volume: all hamsters lost weight after inoculation with SARS-CoV-2. Hamsters inoculated with the standard dose of SARS-CoV-2 in 200 µL volume lost weight faster and to a greater extent than hamsters inoculated with the same dose in 50 µL volume. The difference was statistically significant from four days post-challenge (DPC) onwards (Figure 1a,b). At 6 DPC hamsters had experienced significantly higher (*p* = 0.0004) group mean peak weight loss (Figure 1b), demonstrating more severe disease developing as a result of a higher volume inoculation. No significant difference in weight loss was observed between hamsters inoculated with the standard dose in 50 µL and those inoculated with the 500-fold reduced dose in 200 µL. Qualitative measures of clinical signs of disease excluding weight loss were assigned a score using an arbitrary scale weighted such that clinical signs considered to hold greater clinical significance received higher scores (Table 2) [14] (Figure 1c). An earlier onset of clinical signs was also observed in hamsters inoculated with the standard dose in 200 µL than in hamsters inoculated with the standard dose in 50 µL (Figure 1d–f). There was no significant difference between the groups’ average assigned clinical observation scores.

**Figure 2:**
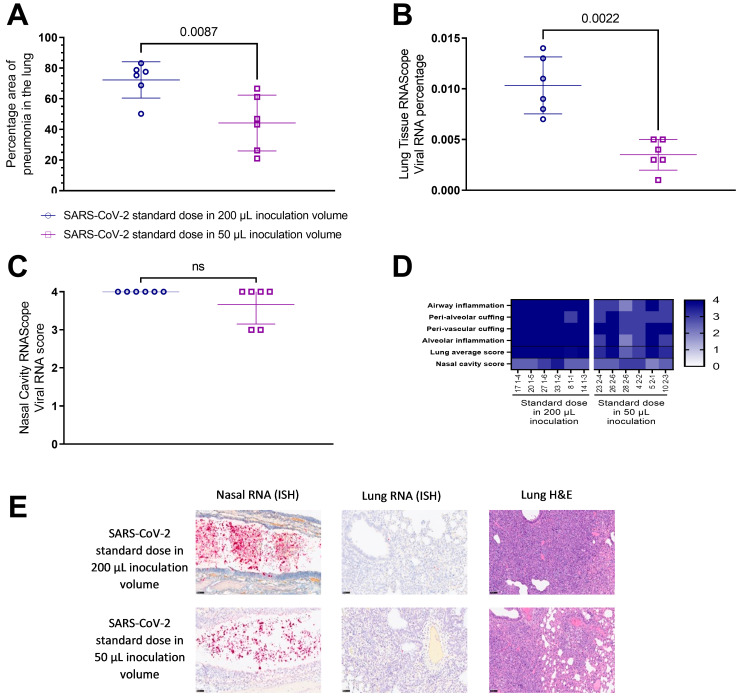
Histopathological findings in the lung and nasal cavity of hamsters challenged with SARS-CoV-2 delivered in varied inoculum volumes 7 days post infection. (**A**) Percentage of areas of pneumonia in the lung determined by image analysis (Nikon-NIS-Ar). (**B**) Percentage of lung positively stained against SARS-CoV-2 RNA measured by in situ-hybridisation (ISH). (**C**) Subjective scores of presence of SARS-CoV-2 RNA in the nasal cavity measured by ISH. (**D**) Heatmap to illustrate severity of histopathological changes in the lung. (**E**) Representative images of viral RNA in the nasal cavity (**left** panels) and lung (**centre** panels) and lung H&E (**right** panels). Statistical differences measured by Mann-Whitney.

Pathological investigation of post-mortem tissues taken at 7 DPC revealed a significantly lower (*p* = 0.0087) total area of pneumonia found in the samples of lung from hamsters inoculated with the standard dose of SARS-CoV-2 (5 × 10^4^ PFU) in a 50 µL total volume (25 µL per nare) compared to those inoculated using a 200 µL total volume (100 µL per nare) (Figure 2a,e). The percentage of the lung containing SARS-CoV-2 RNA as measured by in situ hybridisation also showed significantly lower (*p* = 0.0022) virus in the lung tissue of hamsters inoculated with 50 µL of SARS-CoV-2 than those inoculated with 200 µL (Figure 2b,e). In contrast, the percentage of the nasal cavity containing SARS-CoV-2 RNA as measured by in situ hybridisation showed no significant difference between either inoculation volumes (Figure 2c,e). A marked decrease in pathology scores [19] in the lung tissue of hamsters inoculated with 50 µL of SARS-CoV-2 compared to those inoculated with 200 µL was also observed scoring different parameters of airway and pulmonary parenchyma histopathology (Figure 2d).

**Figure 3:**
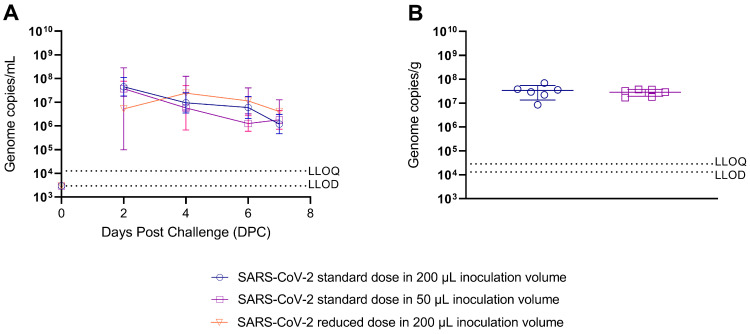
Quantitative polymerase chain reaction (qPCR) analysis of viral shedding and viral load in hamsters challenged with SARS-CoV-2 delivered in varied inoculum volumes. Standard dose of SARS-CoV-2 is 5 × 10^4^ Plaque forming units (PFU)/mL, Reduced dose is 1 × 10^2^ PFU/mL. (**A**) Viral shedding qPCR analysis in throat swab samples taken at 2, 4, 6 and 7 DPC. Lines indicate geomeans and bars indicate 95% confidence intervals. (**B**) Viral load qPCR analysis in lung tissue samples taken 7 DPC. Lines indicate geomeans and bars indicate 95% confidence intervals. No significant difference was detected by one-way ANOVA.

**Viral Shedding titres are unaffected by varied inoculation volumes:** Decreasing either the volume (four-fold) or dose (500-fold) of SARS-CoV-2 inoculum in relation to the standard dose and volume (5 × 10^4^ PFU in 200 µL) did not affect viral shedding titres from the upper respiratory tract (URT) measured in throat swabs (Figure 3a) and no significant differences were found between the amount of total viral RNA shed between groups. Additionally, according to qPCR performed on lung homogenate, we found the viral burden in the lung was unaffected by varying the challenge volume (Figure 3b).

**Figure 4:**
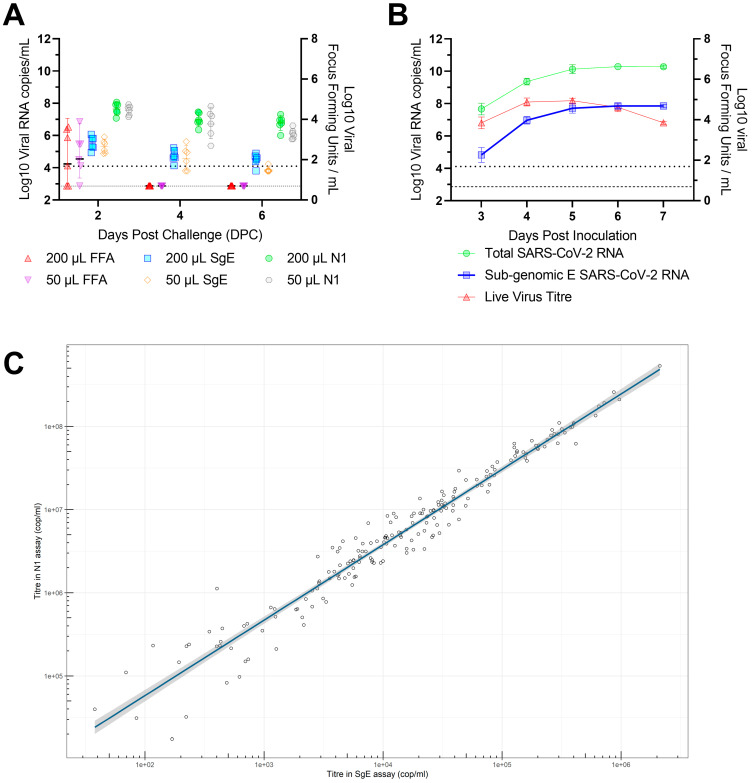
Sub-genomic vs total RNA titre in ex vivo/vitro samples. (**A**) Ex vivo comparison of total genomic viral titre by focus forming assay (FFA), sub-genomic E gene titre by PCR and live viral titre by N1 genomic PCR from 2, 4 and 6 days post challenge (DPC). Dotted line indicates the lower limit of quantification (LLOQ) for sub-genomic and viral PCR values below this are set to half LLOQ for graphing. Dashed line indicates the lower limit of detection (LLOD) for the focus forming assay. (**B**) In vitro comparison of total genomic, subgenomic and live virus titres cultured in Vero-E6 cells. Lines are means and error bars represent standard deviation. (**C**) Comparison of Viral RNA Copies/mL and Sub-genomic RNA copies/mL. Linear regression was used to assess the relationship between total viral RNA copies/mL and sub-genomic RNA copies/mL. The fitted regression model determined a Y-intercept of −2.897, R2 of 0.95 (*p* = 3.81 × 10^−127^).

**Sub-genomic E-gene PCR provides no additional value over total genomic PCR:** Sub-genomic E-gene PCR titres closely correlated with total genomic viral load titres but fall below the limits of quantification and detection too rapidly to accurately compare to live viral titres from ex vivo samples. The correlation between total viral genome copies and sub-genomic E gene copies was highly significant (*p* = 3.81 × 10^−127^), but a high level of variance was observed between live viral titres measured by focus forming assay and both total and sub-genomic RNA titres (Figure 4a). In vitro comparison of total RNA, sub-genomic RNA and live virus titres also demonstrated a very high correlation between total genomic and sub-genomic titres but here live viral titres remained high enough for quantification and showed a decline in live viral titres while both total and sub-genomic titres remained stable (Figure 4b). A large-scale analysis of samples from the CEPI Agility variant screening program [20] also demonstrated a significant linear relationship between total viral RNA titres and sub-genomic RNA titres. The fitted linear regression model determined a Y-intercept of −2.897 indicating three log10 lower sensitivity and an R2 of 0.95 (*p* = 3.81 × 10^−127^) (Figure 4c).

## 4. Discussion

The golden Syrian hamster provides a robust host species in the intranasal model of infection for SARS-CoV-2 to facilitate rapid in vivo analysis of new variants and the efficacy of vaccines, prophylactics and treatments. This species helps to reduce the global scientific communities’ reliance on larger animal models of infection including non-human primates. In this study, we show that changing the volume, whilst maintaining the same viral load, of a SARS-CoV-2 intranasal challenge inoculation significantly alters the severity of disease caused. Hamsters inoculated intranasally with a challenge virus titre of 5.0 × 10^4^ PFU delivered in 50 µL demonstrated a significantly lower peak weight loss–similar to that seen in the animals inoculated with a 500-fold lower dose of SARS-CoV-2. These hamsters also demonstrated markedly reduced severity and slower onset of clinical signs than those intranasally inoculated with the same viral titre in 200 µL. Moreover, histopathological analysis revealed that hamsters receiving the 50 µL inoculation showed significantly lower histopathology scores, smaller areas of pneumonia, and lower viral RNA loads in the lung. No difference was observed in the nasal cavity pathology between hamsters receiving different inoculation volumes. These observations are consistent with the lower volume inoculation not being able to reach as deeply into the lung, remaining mostly in the nasal cavity, an effect previously seen with influenza in ferrets [21]. This may be due to the inoculum being inhaled either intratracheally or as a large particle aerosol and from there entering the lower lung in a similar manner to an aerosol infection and/or directly as a small particle aerosol through inhalation during administration. This suggests that where the virus is able to reach the nasal tract due to direct inoculation, virus replication will occur with similar results. Consideration should be given to whether some of the larger volumes of inoculum may have been swallowed during inoculation making its way into the gastrointestinal tract. It has been shown that ACE2 receptors are found throughout the hamster gut [22]; however, there are conflicting reports on whether oral inoculation with SARS-CoV-2 leads to weight loss and clinical signs in the hamster [23,24]. Intranasal inoculation performed in these studies was performed slowly, allowing each drop of inoculum to enter the nasal cavity prior to the next drop being administered, reducing the likelihood of the inoculum being swallowed. Therefore, these conflicting reports combined with the method in which the inoculum was administered make it unlikely that the increased clinical disease and weight loss in the hamsters receiving the higher volume of inoculum observed here is due to infection via the gastrointestinal tract. 

These data suggest that a larger volume (200 µL, 100 µL in each nare) of intranasal inoculum facilitates an extended viral distribution, faster onset and increased severity of disease. We theorise that the delayed onset of disease, as a result of inoculation with the smaller volume (50 µL, 25 µL in each nare), is due to the initial localised replication of the virus in the upper respiratory tract with later migration towards the lung, at which point weight loss and clinical signs are observed. The effect of inoculum volume on disease outcome is not novel and previous findings with intranasal administration of influenza confirm this. Increased morbidity was observed when mice were administered larger (50 µL or 35 µL) volumes of inoculum intranasally compared to those receiving a smaller volume (25 µL) despite identical doses being given [25]. Miller et al. theorised that the physiological differences in the microarchitecture of the upper and lower respiratory tract contributed to the altered outcome of disease which is likely a contributing factor here too. Ferrets administered with larger volumes of influenza inoculum experienced increased severity of clinical disease and pathology, and it was noted that the larger volume of inoculum was optimal for uniform delivery of the virus to the lower respiratory tract [21]. Cook et al. also reported differences in clinical disease outcomes in mice infected with the same titre of pneumovirus in 10 µL, 25 µL and 50 µL. The severity and duration of the disease were reduced when lowering the volume from 50 µL to 25 µL, and no disease was detected at the 10 µL volume [26].

Despite the reduced severity of disease seen in hamsters receiving the 50 µL inoculum, little difference was seen in viral shedding in the URT compared to the 200 µL inoculated hamsters by qPCR analysis or live viral titre. Reducing the volume of the inoculation did not appear to affect shedding. However, shedding appeared to remain similar between hamsters inoculated with the standard dose and those receiving a reduced infectious dose, suggesting that viral loads measured by PCR lack sensitivity to detect alterations in shedding. Viral load qPCR in the lung also demonstrated no difference between the two inoculum volumes at the post-mortem at 7 DPC. This is particularly interesting as the viral load measured by in situ hybridisation showed significantly lower RNA levels in the lungs of the 50 µL inoculated hamsters. This apparent mismatch between results may be due to limitations of the qPCR assay or the in situ hybridisation method and warrants further investigation. Sub-genomic RNA has been suggested as a way to determine actively replicating viruses as the leader sequence is believed to only be added to replicating RNA [27,28]. The absolute mechanism of coronavirus replication and the involvement of the sub-genomic RNA remains unresolved [29,30]. A very strong relationship between total viral RNA and sub-genomic RNA was identified, with sub-genomic RNA titre forming an almost 1000-fold lower titre subpopulation of total viral RNA. This indicates a simple scalar relationship between them. Further, live viral titres were undetectable two days after challenge, while sub-genomic RNA remained detected until the end of the study at seven days post-challenge. Sub-genomic PCR does not provide a specific indicator of live virus titres consistent with other studies [31,32] and provides no additional information beyond that of the total genomic RNA titre. 

The procedural details of the inoculation are likely to enhance the chance of inhalation into the lung parenchyma. The enhanced severity of disease provided by using a 200 µL volume for intranasal inoculation of our standard dose (5.0 × 10^4^ PFU) has helped us to identify differences in pathogenicity between variants as well as better distinguish the efficacy of vaccines, therapeutics and therapies [13,19]. A further infection study in hamsters comparing inhalational administration of a small particle aerosol with our standard intranasal volume and dose would be a useful further investigation to confirm this hypothesis.

## Figures and Tables

**Table 1:** An illustration of the variation in intranasal challenge inoculum volume found in the literature. This is a representative sample and non-exhaustive.

Publication	Volume of Intranasal Inoculation (µL)	Specifies per Nare?
Rosenke et al. [3]	50 total (25 per nare)	Yes
Abdelnabi et al. [10]	50 total (25 per nare)	Yes
Kawaoka et al. [9]	30 total	No
Osterrieder et al. [11]	60 total	No
Yuan et al. [12]	100 total	No
Yamasoba et al. [8]	100 total	No
Huo et al. [13]	200 total (100 per nare)	Yes
Ryan et al. [14]	200 total (100 per nare)	Yes
Song et al. [15]	200 total	No
Zhao et al. [16]	Only PFU provided—cites paper using 100 µL per nare	No
Sia et al. [17]	Only PFU provided	No

**Table 2:** Scoring system for clinical observations.

Clinical Sign	Score
Healthy	0
Ruffled fur	1
Wasp-waisted	3
Laboured breathing	5
Severe laboured breathing	7

## Data Availability

Not Applicable.
